# Supplementary material for: Structure and dynamics of an archetypal DNA nanoarchitecture revealed via cryo-EM and molecular dynamics simulations
Source: Nat Commun. 2023 Jun 19;14:3630. doi: 10.1038/s41467-023-38681-5 (PMC10279742; doi:10.1038/s41467-023-38681-5)
Supplement: Supplementary file 1 — Supplementary Information [file 41467_2023_38681_MOESM1_ESM.pdf]

Supplementary Information

Structure and dynamics of an archetypal DNA nanoarchitecture revealed via cryo-EM and molecular dynamics simulations

1. Supplementary Tables and Figures

Supplementary Table 1. Names, chemical modifications, and sequences of DNA oligonucleotides used to prepare the 6HB DNA nanostructure.

ID	Sequence from 5′ to 3′
1	AGCGAACGTGGATTTTGTCCGACATCGGCAAGCTCCCTTTTTCGACTATT
2	CCGATGTCGGACTTTTACACGATCTTCGCCTGCTGGGTTTTGGGAGCTTG
3	CGAAGATCGTGTTTTTCCACAGTTGATTGCCCTTCACTTTTCCCAGCAGG
4	ATCAACTGTGGTTTTTCTCACTGGTGATTAGAATGCTTTTGTGAAGGGC
5	CACCAGTGAGATTTTTGTCGTACCAGGTGCATGGATTTTTGCATTCTAA
6	CCTGGTACGACATTTTTCCACGTTCGCTAATAGTCGATTTTATCCATGCA
1(chol)	Sequence of 1, carries a cholesterol via a tri(ethylene glycol) linker at the 3' terminus
3(chol)	Sequence of 3, carries a cholesterol via a TEG linker at the 3' terminus
5(chol)	Sequence of 5 carries a cholesterol via a TEG linker at the 3' terminus

Supplementary Table 2. DNA oligonucleotides composition of 6HB used for cryo-EM and molecular dynamics simulations nanostructure.

Method	Composition
Cryo-EM	1, 2, 3, 4, 5, 6
MD	1(chol), 2, 3(chol), 4, 5(chol), 6

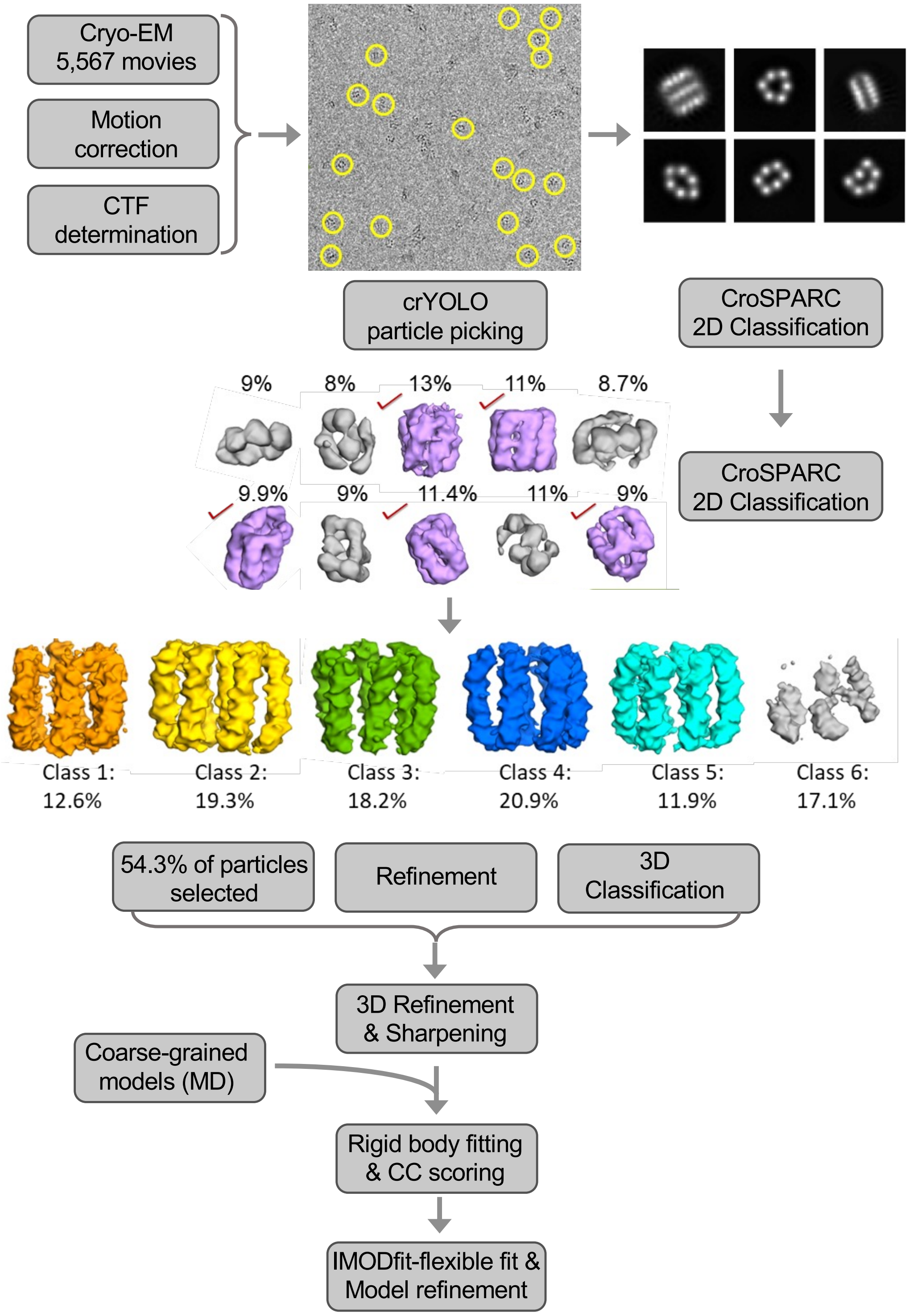

Supplementary Figure 1. Cryo-EM workflow outlining the process of obtaining a structure.

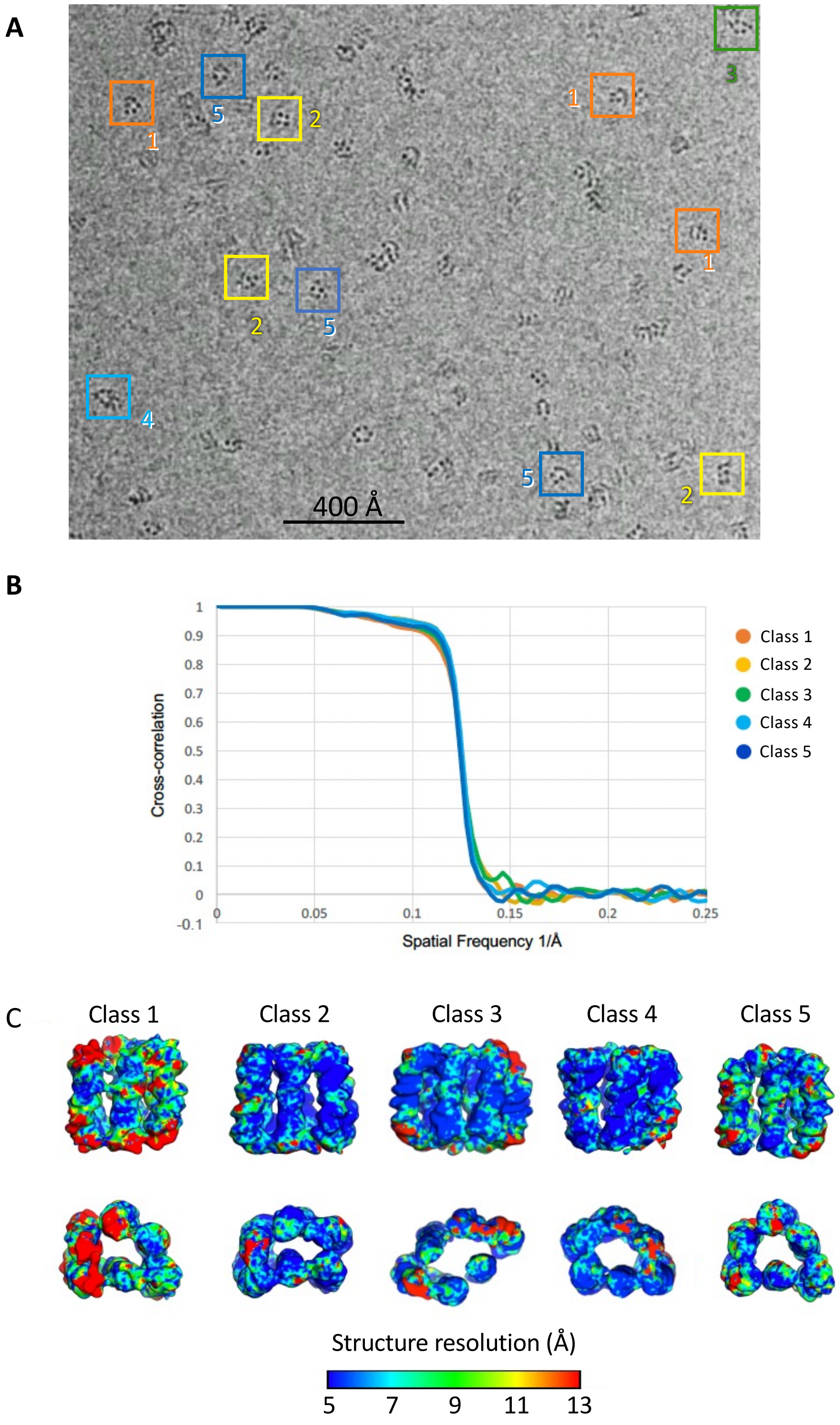

Supplementary Figure 2. Cryo-EM structural resolution attainment. A) Representative micrograph. **B)** Fourier-shell correlation curves of the final 3D maps of structural classes 1-5. **C)** Local resolution maps for each of the five structural classes of 6HB shown both top-down and side-on. The maps are coloured to indicate the local resolution in Å.

Supplementary Table 3. Cross-correlation score and RMSD for the fits of the models to cryo-EM maps

EM Class	MD model (0.3M NaCl)	Rigid body fit (CC)	Flexible fit (CC)	RMSD (Å) Rigid-body vs flexible fit
1	9	0.82	0.90	6.0
2	1	0.81	0.91	6.7
4	5	0.80	0.91	5.56
5	9	0.81	0.87	6.14
6	9	0.83	0.91	4.9

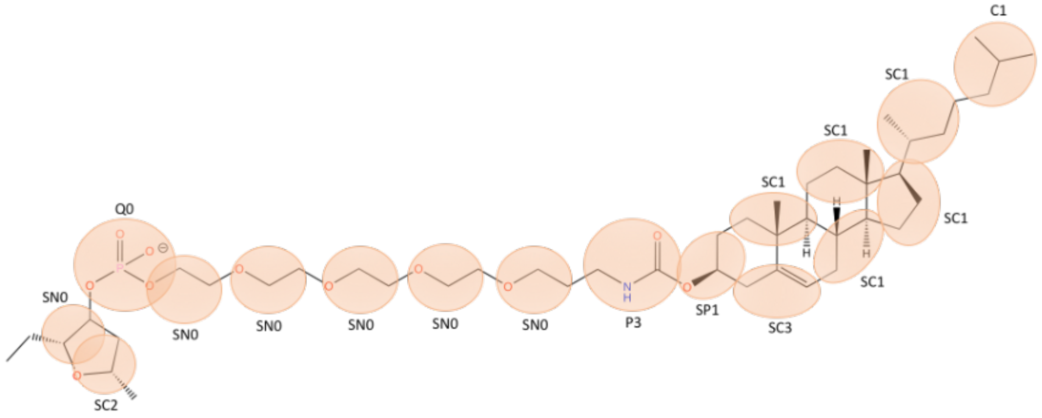

Supplementary Figure 3. Atom-to-CG mapping used to generate MARTINI parameters for the TEG-C anchors.

Supplementary Table 4. Summary of MD simulations

System	Number of CG replicas	Duration of CG replicas	Number of AA replicas‡	Duration of AA replicas
0.3 M NaCl (no bilayer)	15	500 ns	21	30 ns
1.0 M NaCl (no bilayer)	15	500 ns	-	-
0.3 M NaCl (with bilayer)	15	1 μs	-	-
1.0 M NaCl (with bilayer)	15	1 μs	-	-

‡ Six of these AA replicas were used to generate the CG parameters for the TEG-cholesterol anchors, before the CG replicas were run. The remainder of the AA replicas were simulated after the CG simulations were completed, for validation of the results obtained for the solvated CG nanopore model in 0.3 M NaCl.

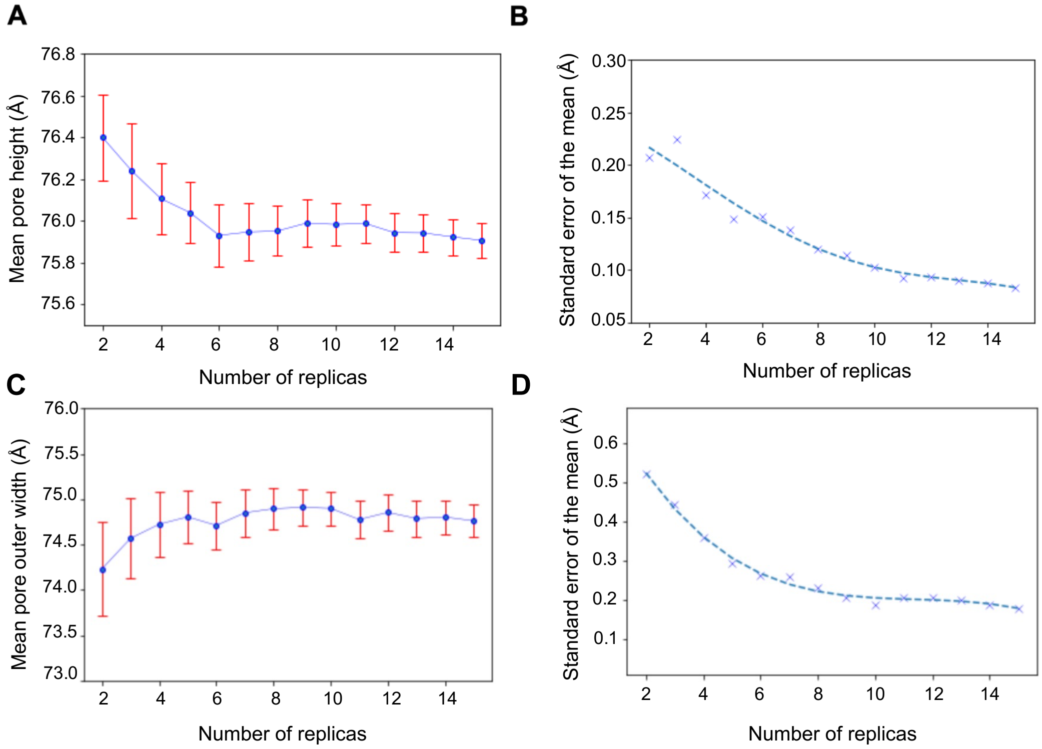

Supplementary Figure 4. Plots illustrating the convergence of 6HB dimensions and the standard errors of the mean with increasing number of simulations. The stabilisation of mean dimensions around a mean as well as the decay of the associated standard errors indicates that fifteen simulation replicas are sufficient to ensure reproducible results. Error bars correspond to the bootstrapped standard error of the mean. **A)** Convergence of the mean pore height as a function of replica number. **B)** Decay and subsequent plateau of the standard error associated with the pore height. **C)** Convergence of the mean pore outer width as a function of replica number. **D)** Decay of the standard error associated with the pore outer width. The simulations were conducted in 0.3 M NaCl.

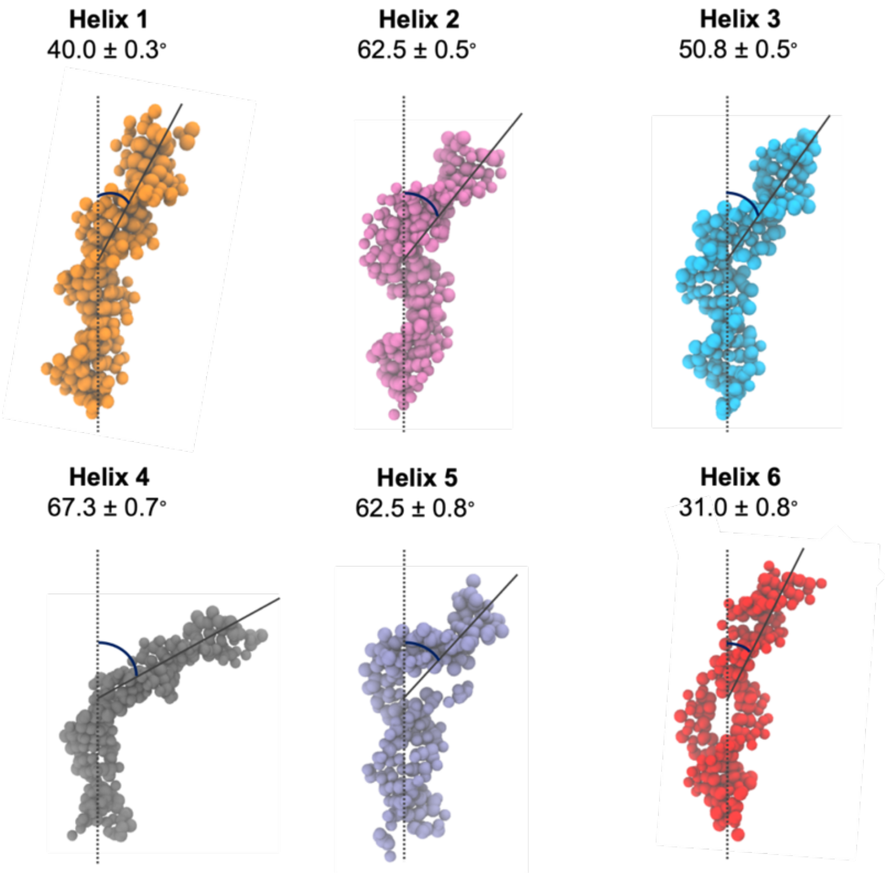

Supplementary Figure 5. Kink angles for each of the six duplexes calculated from 15 CG simulation trajectories of 6HB in 0.3 M NaCl. The errors correspond to the bootstrapped standard error of the mean.

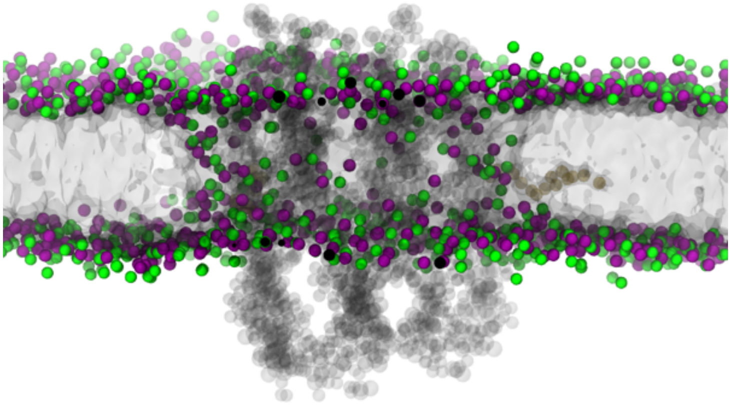

Supplementary Figure 6. Snapshot from a simulation trajectory of 6HB embedded in a POPC membrane in 0.3 M NaCl. The snapshot illustrates how the tilting of the charged POPC headgroups (choline moieties in green, phosphate groups in purple) leads to formation of a lipid torus around the nanopore. Only one of the cholesterol anchors (orange) is shown to enhance visibility of the lipid torus.

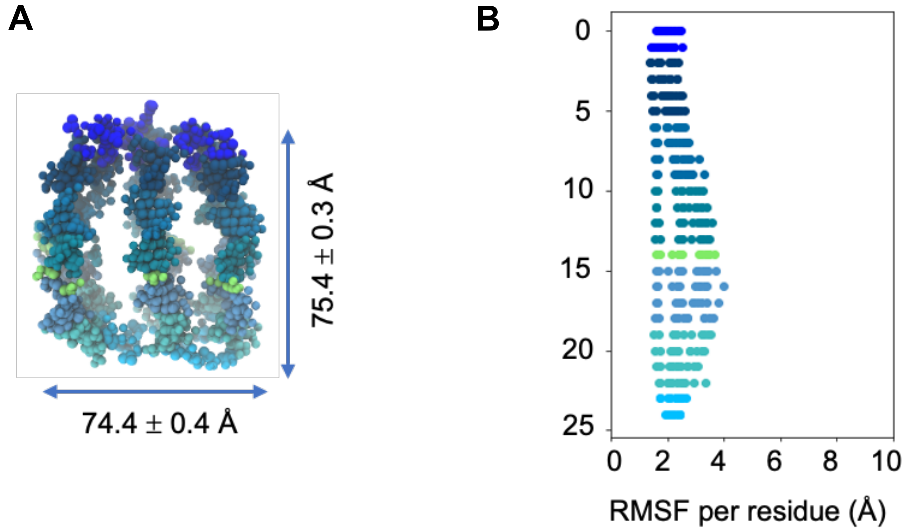

Supplementary Figure 7. Per-residue root-mean-squared fluctuation of 6HB solvated in 1.0 M NaCl. A) Snapshot of 6HB simulated in a solution of 1.0 M NaCl with the average pore height and width. Errors represent the standard error of the mean. **B)** Root-mean-squared fluctuation (RMSF) per-residue plot obtained from the simulation of 6HB solvated in 1.0 M NaCl. Each dot represents a single nucleotide, and is coloured according to its position along the length of the 6HB as shown in A.

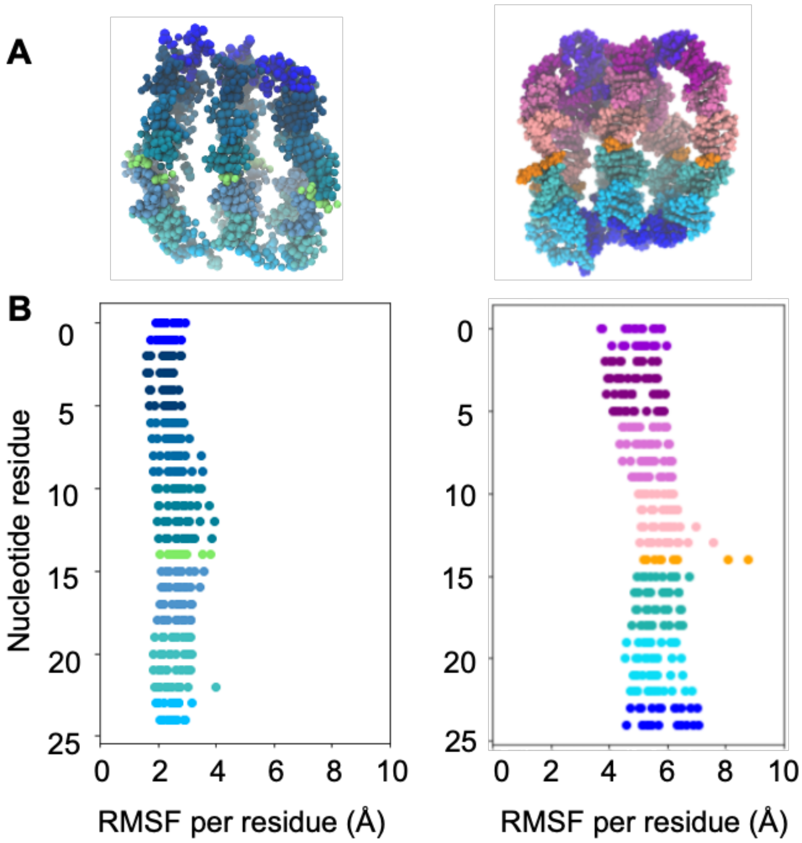

Supplementary Figure 8. A comparison of the root-mean-squared fluctuations of the CG model to the reference AA model. A) Representative structures of the CG (left) and AA (right) models of the 6HB outside of the membrane, in 0.3 M NaCl. **B)** The corresponding RMSF-per-residue plots. The AA RMSF-per-residue plot (right) was generated from an ensemble of 15 x 30 ns AA simulations of the 6HB outside of the membrane in 0.3 M NaCl. The plot on the left corresponds to Figure 5B in the main manuscript, displaying the RMSF-per-residue obtained from the CG simulation ensemble of the 6HB outside of the membrane in 0.3 NaCl, while the plot on the right displays the equivalent generated from reference AA simulations. Overall, the median RMSF values for each residue in the AA model are around twice the values seen in the CG model, indicating that the conformational flexibility of the 6HB is supressed in the CG model. This is likely a consequence of the stiff elastic network model used in the CG simulations, which applies flexible restraints to all the pseudo-atom beads in order to preserve the B-form structure.

Supplementary Table 5. Base-pair and base-step parameters calculated from the ensemble of 15 x 30 ns AA simulations of the 6HB outside on the membrane in 0.3 M NaCl. Errors correspond to the standard error of the mean. Literature values for the known idealised parameters for B- and A- forms of DNA are also listed for comparison[1,2].

	Stretch (Å)	Shear (Å)	Stagger (Å)	Buckle (∘)	Opening (∘)	Propeller (∘)	Rise (Å)	Twist (∘)	Roll (∘)	Tilt (∘)	Shift (Å)	Slide (Å)
**Helix 1**	-1.15 ± 0.30	0.381 ± 0.240	-0.440 ± 0.196	0.478 ± 0.519	0.017 ± 1.450	-10.7 ± 0.8	3.28 ± 0.05	30.4 ± 1.3	5.93 ± 0.75	-2.58 ± 1.35	-0.128 ± 0.070	0.465 ± 0.094
**Helix 2**	1.02 ± 0.31	-0.023 ± 0.145	-0.202 ± 0.380	0.0967 ± 0.629	3.14 ± 2.61	-7.75 ± 0.69	3.54 ± 0.13	34.3 ± 2.25	2.53 ± 0.34	-0.438 ± 0.637	0.839 ± 0.084	-0.455 ± 0.226
**Helix 3**	-0.219 ± 0.096	0.133 ± 0.122	0.0835 ± 0.0579	-0.885 ± 0.404	1.60 ± 0.71	-10.3 ± 0.6	3.46 ± 0.03	34.5 ± 1.3	5.06 ± 0.35	1.38 ± 0.67	0.174 ± 0.066	0.603 ± 0.078
**Helix 4**	-0.585 ± 0.100	-0.521 ± 0.105	-0.751 ± 0.111	-0.595 ± 0.099	2.80 ± 1.39	-3.99 ± 0.65	4.16 ± 0.209	30.9 ± 1.1	1.69 ± 0.91	-2.78 ± 0.82	-0.099 ± 0.204	1.29 ± 0.24
**Helix 5**	-0.405 ± 0.065	0.0106 ± 0.0332	-0.506 ± 0.060	-1.49 ± 0.35	-0.501 ± 0.522	-10.8 ± 0.4	3.55 ± 0.03	35.6 ± 0.2	2.95 ± 0.38	1.46 ± 0.14	-0.121 ± 0.026	0.680 ± 0.068
**Helix 6**	1.102± 0.065	-0.011 ± 0.033	-0.202 ± 0.383	0.097 ± 0.632	3.14 ± 2.56	-7.75 ± 0.69	1.71 ± 0.34	22.7 ± 2.2	2.53 ± 0.34	-0.438 ± 0.639	-0.121 ± 0.026	0.839 ± 0.085
**B-DNA (ideal)**	~0	~0	~0	± 20 (REF[2])	~0	-10 (REF[2])	3.4 (REF[3])	36 (REF[3])	---	-6 to -16 (REF[3])	~0	~0
**A-DNA (ideal)**	---	---	---	---	---	---	2.6 (REF[2])	33 (REF[2])	---	10 to 20 (REF[2])	---	---

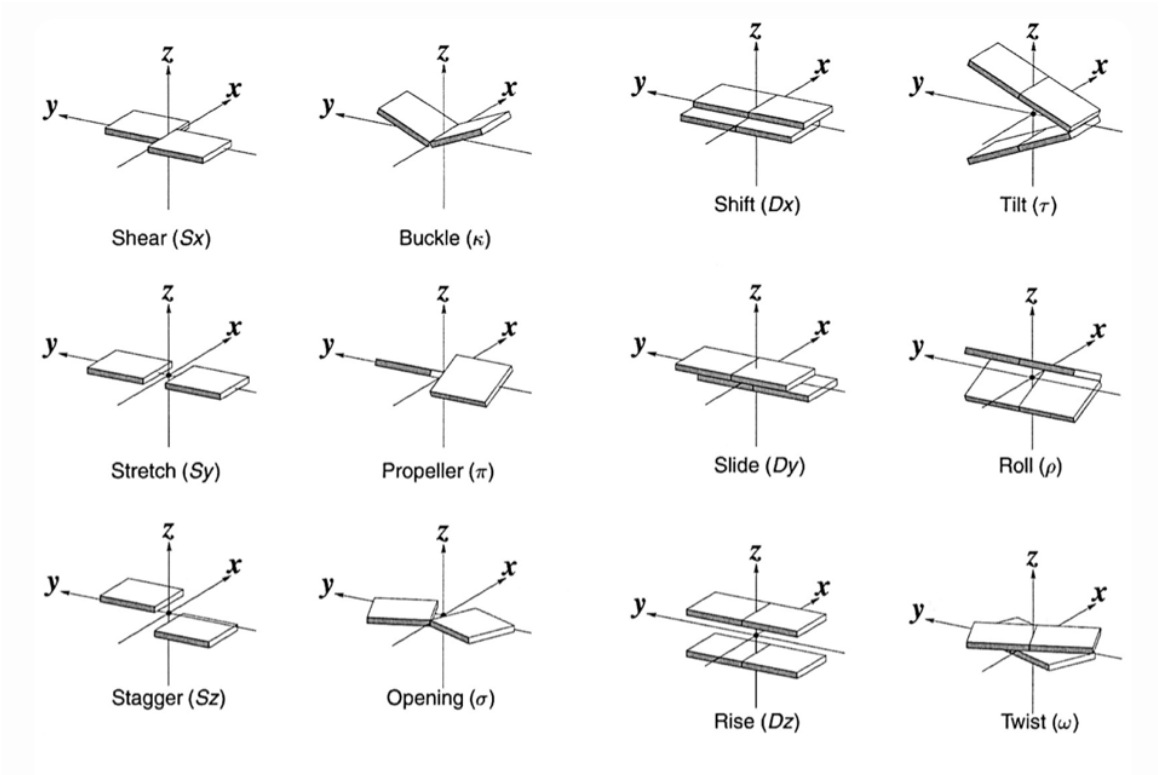

Supplementary Figure 9. Pictorial representations of the six base-pair parameters (shear, stretch, stagger, buckle, propeller and opening) and the six base-step parameters (shift, slide, rise, tilt, roll, twist). These parameters were calculated from the AA simulation ensemble of the of the 6HB in 0.3 M NaCl. Figure adapted with permission from Ref.[2].

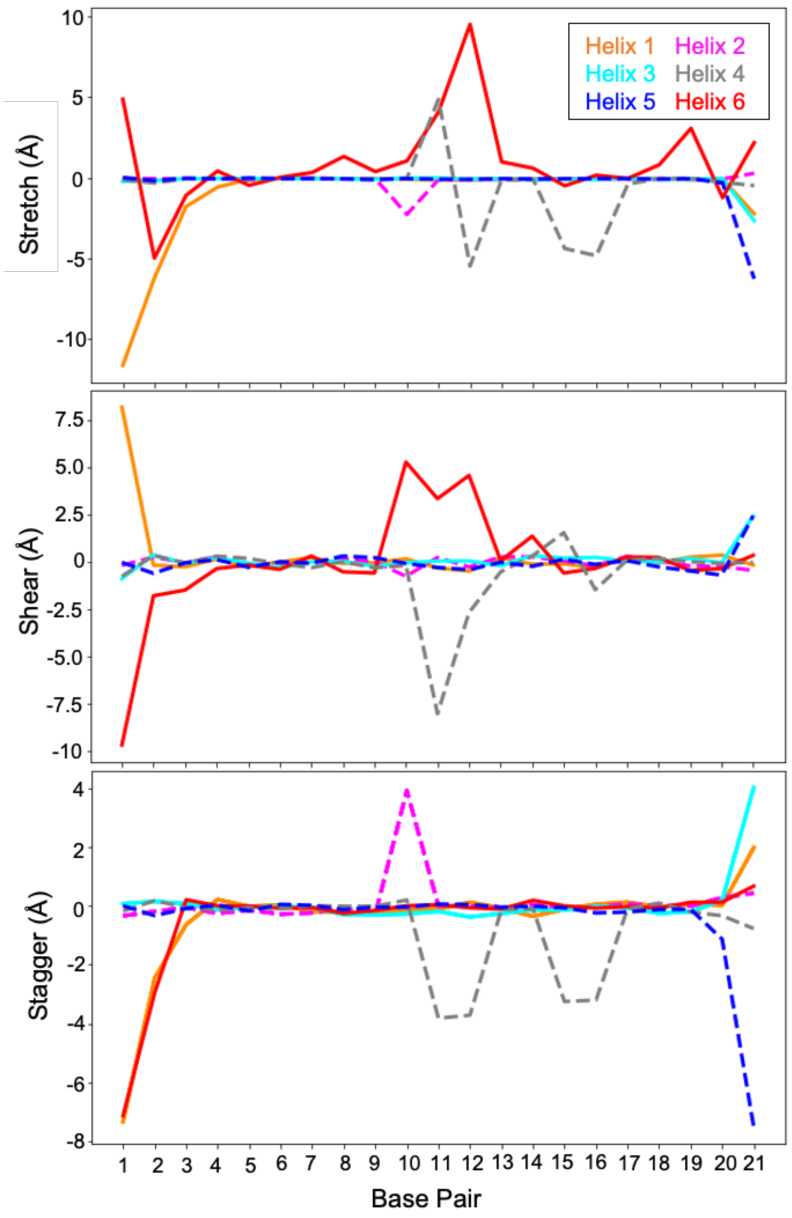

Supplementary Figure 10. Plots showing the variation of the ensemble-averaged stretch, shear, and stagger parameters along the base-pairs of each helix. Base pairs 1 to 12 correspond to the base pairs present in the longer helix sections comprised of residues 1 to 14, while base pairs 13 to 21 correspond to the base pairs present within the shorter helix sections (residues 15 to 25). The nick site lies between base pairs 12 and 13. Extreme negative stretch values such as those observed in helices 1, 4 and 6 indicate base-pair breakage via base-flipping[4]. Helices 1, 3 and 4 exhibit extreme base-pair shearing at the helix crossovers the top of the 6HB, and near the nick sites. Significant base-pair staggering is observed in all helices but is generally localised to the vicinity of the nick sites and the terminal crossovers.

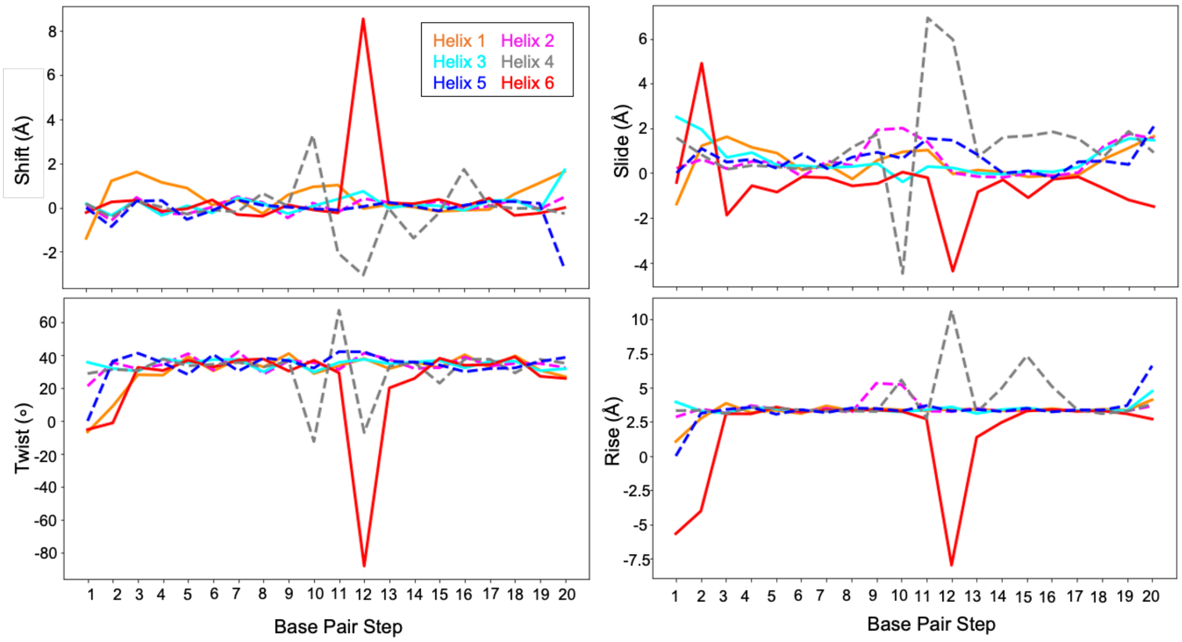

Supplementary Figure 11. Plots showing the variation of the ensemble-averaged shift, slide, twist and rise parameters along the base-steps of each helix. In an idealised model of base-stacking in B-DNA, shift and slide should be close to zero, while twist and rise should stay near their typical values of ~ 36° and 3.4 nm, respectively. All six helices experience some deviation from the idealised values, though helices 1 4 and 6 are the most affected, due to the strain at the helix crossovers at the termini and fraying at the nicks which causes some bases to flip out and turn over, giving rise to unusually extreme values for these parameters.

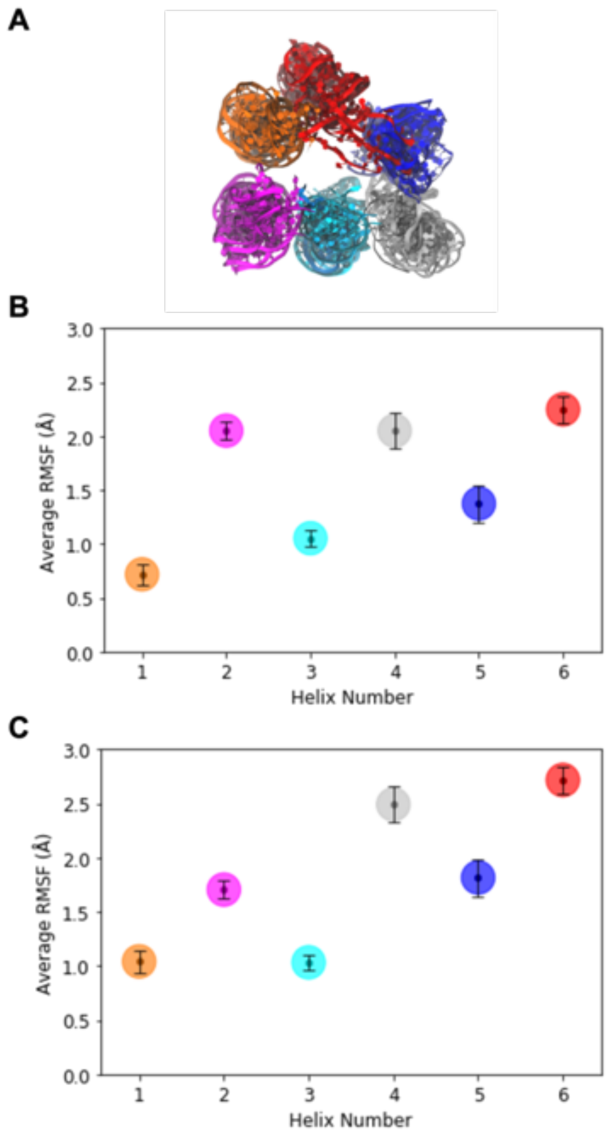

Supplementary Figure 12. Per-duplex root-mean-squared fluctuation analysis of 6HB in low and high salt conditions. A) Snapshot of the simulated 6HB viewed top-down with each duplex uniquely coloured. **B)** RMSF per-duplex plot obtained from the simulation of the 6HB solvated in 0.3 M NaCl. The points for each helix are coloured to match the plot in A. **C)** RMSF per-duplex plot obtained from the simulation of the 6HB solvated in 1.0 M NaCl. The points for each helix are coloured to match the plot in A. The RMSF is calculated for the entire ensemble of configurations sampled in the set of simulations. At every simulation time-step (i.e. every 5 fs of simulation time) the atomic positions are updated, and the configuration changes. The RMSF is therefore a time-averaged standard deviation of the atomic positions with respect to a reference frame. Error bars represent the standard deviation of averaged RMSF values which were calculated for each individual simulation trajectory.

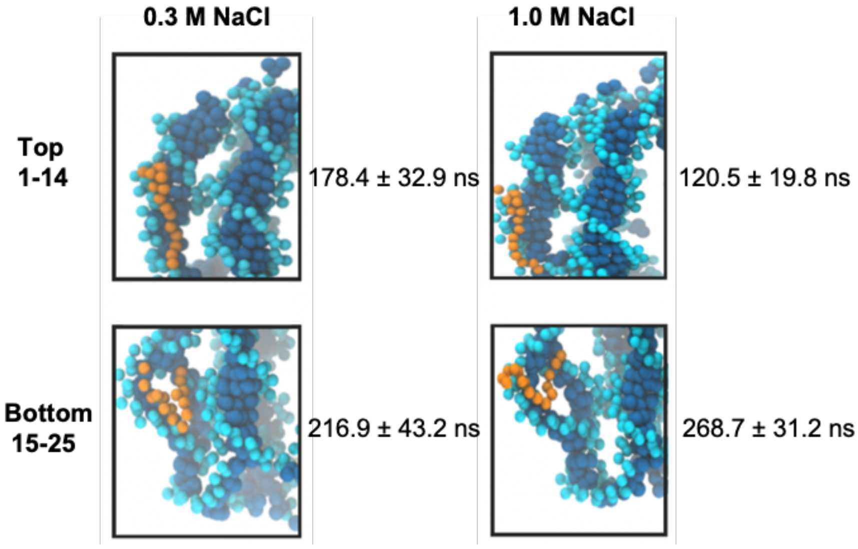

Supplementary Figure 13. Interaction between the cholesterol tag and DNA duplex in 0.3 M and 1.0 M NaCl. Simulations in solution show that the cholesterol lipid anchor non-covalently interacts with the 6HB duplex it is chemically attached to. The interaction with the DNA groove at the top (above the nick) or the bottom (below the nick) of 6HB. This interaction is measured by the average dwell time of the cholesterol tag within the groove of the DNA duplex in both regions at two different salt conditions. Errors correspond to the standard error of the mean.

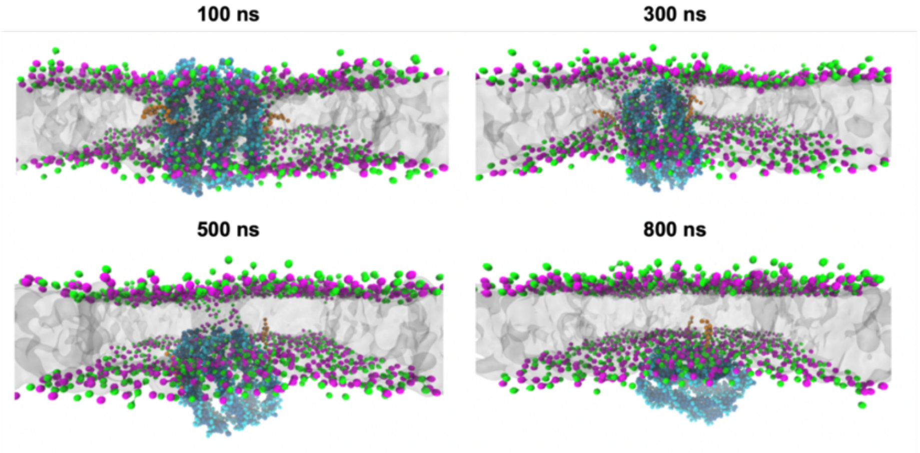

Supplementary Figure 14. Simulation snapshots showing the multi-step process of 6HB membrane-ejection in 0.3 M NaCl taken from one simulation trajectory. At 100 ns, 6HB adopts a transmembrane orientation within the POPC lipid bilayer. At 300 ns, a reorganisation of the membrane lipids occurs causing 6HB to drift towards the bottom of the membrane. The pore no longer spans the membrane. At 500 ns, 6HB tilts and rotates. The two bilayer leaflets are no longer continuous. At 800 ns, 6HB moves completely out of the membrane and adopts a membrane-tethering orientation of the membrane surface.

Supplementary Table 6. Mean number of sodium and chloride ions residing within the pore lumen in the two investigated salt conditions, calculated from the two simulation ensembles. Errors correspond to the standard error of the mean.

NaCl concentration (M)	Mean number of Na^+ ions in pore lumen	Mean number of Cl^- ions in pore lumen
0.3 M	180.1 ± 1.7	7.7 ± 0.4
1.0 M	264.5 ± 2.2	66.9 ± 1.9

2. Supplementary Videos

Supplementary Video 1: Coarse-grained 6HB simulated in 0.3 M NaCl. This trajectory slice represents 30 ns of simulation time.

Supplementary Video 2: Coarse-grained 6HB simulated in 1.0 M NaCl. This trajectory slice represents 30 ns of simulation time.

Supplementary Video 3: Coarse-grained 6HB simulated in a POPC bilayer in 0.3 M NaCl solution. The clip corresponds to the first 100 ns of a single trajectory, sped up by a factor of 4.

Supplementary Video 4: Coarse-grained 6HB simulated in a POPC bilayer in 1.0 M NaCl solution. The clip corresponds to the first 100 ns of a single trajectory, sped up by a factor of 4.

Supplementary Video 5: Expulsion of 6HB from the POPC bilayer in 0.3 M NaCl solution. The clip is taken from a trajectory that featured an expulsion event, and corresponds to 400 ns of simulation time, sped up by a factor of 20.

3. Supplementary References

1. Ussery, D. W. DNA structure: A-, B- and Z-DNA helix families. in *eLS* (Wiley, 2002).

2. Lu, X.-J. & Olson, W. K. 3DNA: A software package for the analysis, rebuilding and visualization of three-dimensional nucleic acid structures. *Nucleic Acids Res.* **31**, 5108–5121 (2003).

3. Calladine, C. R., Drew, H. R., Luisi, B. F. & Travers, A. A. *Understanding DNA: The molecule and how it works: Third edition* (Elsevier Ltd, 2004).

4. Bouvier, B. & Grubmüller, H. A Molecular dynamics study of slow base flipping in DNA using conformational flooding. *Biophys. J.* **93**, 770–786 (2007).
