# Supplementary material for: Structure and dynamics of an archetypal DNA nanoarchitecture revealed via cryo-EM and molecular dynamics simulations
Source: Nat Commun. 2023 Jun 19;14:3630. doi: 10.1038/s41467-023-38681-5 (PMC10279742; doi:10.1038/s41467-023-38681-5)
Supplement: Supplementary file 3 — Description of Additional Supplementary Files [file 41467_2023_38681_MOESM3_ESM.pdf]

Description of Additional Supplementary Files

File Name: Supplementary Movie 1
Description: Coarse-grained 6HB simulated in 0.3 M NaCl. This trajectory slice represents 30 ns of simulation time.

File Name: Supplementary Movie 2
Description: Coarse-grained 6HB simulated in 1.0 M NaCl. This trajectory slice represents 30 ns of simulation time.

File Name: Supplementary Movie 3
Description: Coarse-grained 6HB simulated in a POPC bilayer in 0.3 M NaCl solution. The clip corresponds to the first 100 ns of a single trajectory, sped up by a factor of 4.

File Name: Supplementary Movie 4
Description: Coarse-grained 6HB simulated in a POPC bilayer in 1.0 M NaCl solution. The clip corresponds to the first 100 ns of a single trajectory, sped up by a factor of 4.

File Name: Supplementary Movie 5
Description: Expulsion of 6HB from the POPC bilayer in 0.3 M NaCl solution. The clip is taken from a trajectory that featured an expulsion event, and corresponds to 400 ns of simulation time, sped up by a factor of 20.
